# Stress-induced cortisol is associated with generation of non-negative interpretations during cognitive reappraisal

**DOI:** 10.1186/s13030-015-0049-x

**Published:** 2015-11-07

**Authors:** Hideki Tsumura, Jun Sensaki, Hironori Shimada

**Affiliations:** Faculty of Medicine, Shimane University, 89-1 Enya-cho, Izumo-shi, Shimane 693-8501 Japan; Graduate School of Human Sciences, Waseda University, 2-579-15-101-321 Mikajima, Tokorozawa, Saitama 359-1192 Japan; Faculty of Human Sciences, Waseda University, 2-579-15-101-321, Mikajima, Tokorozawa, Saitama 359-1192 Japan

**Keywords:** Cortisol, Cognitive reappraisal, Stress, Interpretation, Depressive mood

## Abstract

**Background:**

Enhanced processing of emotional stimuli after stress exposure is reported to be associated with stress-induced cortisol. Because enhanced emotional information processing could make cognitive emotion regulation more difficult, it was hypothesized that stress-induced cortisol would be associated with non-negative interpretation generation associated with the cognitive reappraisal processes.

**Methods:**

A total of 36 participants (Mean age = 21.3 years, *SD* = 1.8) watched video clips of depression-related stressful situations before and after the administration of a stress induction task. They were then asked to generate as many non-negative interpretations as possible to reduce the depressive mood. Saliva samples were obtained before and after the stress induction task to measure change in the cortisol level.

**Results:**

Participants were allocated post-hoc to either a responder (*n* = 19) or non-responder group (*n* = 17) based on the cortisol response to the stress induction task. The number of non-negative interpretations generated following the stress induction task was reduced only in the cortisol responders. The number of post-stress non-negative interpretations was fewer in the responder group when compared by sex, baseline cortisol level, and the number of pre-stress non-negative interpretations, statistically controlled.

**Conclusions:**

Although baseline cortisol and sex may have impacted the results, the results suggest that stress-induced cortisol is associated with difficulty in non-negative interpretation generation during the cognitive reappraisal process.

## Background

Enhanced processing of negative stimuli contributes to high negative affective response, enduring negative affect, and emotional disorders [1]. It involves enhanced selective attention toward a negative stimulus relative to a neutral stimulus (i.e., attentional bias) and enhanced encoding, storage, and retrieval of negative stimuli (i.e., memory bias). Enhanced processing of negative stimuli is observed under acute psychosocial stressors [2]. Stressors induce cortisol release, which is the final hormone of the hypothalamic–pituitary–adrenal (HPA) axis. Change in emotional information processing after stress exposure is reported to be associated with stress-induced cortisol [3], and stress-induced cortisol is assumed to be one of the possible biological mediators between stress and enhanced emotional information processing. The brain is one of the targets of cortisol, and the prefrontal cortex, hippocampus, and amygdala have a high density of corticosteroid receptors [4, 5]. It is assumed that cortisol could modulate emotion information processing through its actions in these areas.

Enhanced processing of emotional stimuli could make cognitive emotion regulation more difficult. Emotion regulation refers to shaping the kind of emotions that one possesses and their strength [6]. Cognitive emotion regulation involves the alternation of ongoing emotional responses through the processing of emotional stimuli. Two types of cognitive emotion regulations have been identified, attentional control and cognitive change [7]. Attentional control is implemented at an early information processing stage immediately following sensory intake and includes selective inattention toward negative stimuli. Cognitive change is implemented at a later and more elaborate information processing stage and includes cognitive reappraisal, which is defined as reinterpreting a stimuli or a situation in such a way as to reduce its emotional impact [8]. Cognitive change processes involve generating and retaining non-negative interpretations while inhibiting negative interpretations of stimuli or situations that are evaluated as stressful [9]. Therefore, enhanced information processing of negative stimuli might interfere with these cognitions.

Stress-related psychiatric disorders that include aspects of depression are related to the absence of effective use of cognitive emotion regulation [10]. In addition, individuals with depression have been shown to generate fewer non-negative interpretations of stressful social situations [11]. Cortisol is one of the neurobiological correlates of depression [12], which may be partly due to the cortisol action on generation of non-negative interpretations. Although previous studies have not investigated the relation between stress-induced cortisol and cognitive reappraisal, stress-induced cortisol may be associated with impairment in the later information processing stage as well as with impairment in the earlier stage that includes attentional bias [3].

This study aimed to investigate the relationship between stress-induced cortisol and non-negative interpretations generated to regulate depressive mood in response to depression-related stimuli. We compared changes in the generation of non-negative interpretations before and after stress induction of participants with and without cortisol elevation in response to stress induction (responders and non-responders) to examine differences in their information processing of depression-related stimuli between conditions with and without cortisol elevation. We tested the hypotheses that the responders would generate fewer non-negative interpretations than the non-responders, for whom the number would be constant.

## Methods

### Participants

Thirty-six (25 female and 11 male) undergraduate or graduate students with a mean age of 21.3 years (*SD* = 1.8) participated in the present study. The participants were recruited through poster advertisements and announcements on a university campus. Exclusion criteria were a self-reported history of smoking, current psychiatric disorders, and use of any medication or alcohol consumption on the day of the experiment. The study was approved by the university ethics committee. All the participants signed a written informed consent form and were paid for their participation.

### Measures

#### Depressive symptoms

Depressive symptoms were measured using the Japanese version of the Center for Epidemiologic Studies-Depression Scale (CES-D) [13], originally developed by Radloff [14]. The CES-D consists of 20 items rated on a 4-point Likert scale, with the scores ranging from zero to 60, as in the original version. The Japanese version of the CES-D has been demonstrated to be reliable and valid [13]. The CES-D was used to compare the levels of depressive symptoms of responders and non-responders.

#### Negative affect

Negative affect was measured using the negative affect subscale of the Japanese version of the Positive and Negative Affect Schedule (PANAS) [15], originally developed by Watson, Clark, & Tellegen [16]. The PANAS is a self-rating scale comprising two subscales that measure positive and negative affect. Two items in the original version were omitted from the negative affect subscale of the Japanese version of the PANAS (PANAS-N) due to low communality in factor analysis [15]. Thus, the Japanese version of PANAS-N consists of eight items, such as “distressed” and “nervous”. Current negative affect was rated on a 6-point Likert scale, with the scores ranging from 8 to 48. The Japanese version of the PANAS has been demonstrated to be reliable and valid [15]. The PANAS-N was used to confirm that the stress induction task used in the study enhanced negative affect.

#### Cortisol levels

Saliva samples were collected between 1345 and 1620 h because the diurnal variation of cortisol is lower in the afternoon. Saliva sampling was conducted by passive drool, i.e., participants were asked to draw saliva into their mouth for two minutes, and then drool into a specimen tube through a 4-cm-long straw. Saliva samples were stored in a freezer below −20 °C between collection and assay. The cortisol level was measured by means of an enzyme-linked immunosorbent assay (ELISA), using a commercial kit (Salimetrics, State College, PA, USA). The inter- and intra-assay variances were 4.96 and 4.43 %, respectively.

### Psychological stress induction task

A mental arithmetic task was used for psychological stress induction. Mental arithmetic tasks have been confirmed to elicit negative affect and cortisol responses [17]. Participants were asked to serially subtract out loud 17 from 3093 for 5 min, as quickly and accurately as possible. They calculated in front of both a video recorder and a judge of the same sex as the participant and were informed that their task performance was recorded by the video and evaluated by the judge. The judge asked participant to restart the calculation in the case of miscalculations.

### Experimental task

Four kinds of video clips of depression-related stressful social situations, one for practice and three for measurement, were created for the present study. It has been reported that viewing negative stimuli does not induce cortisol release [18], thus viewing video clips of depression-related situations would not induce cortisol release. The scenarios of the video clips for measurement were based on the scenarios from the Cognitive Bias Questionnaire (CBQ) [19]. The scenarios were 1) encouraged by your friends, you run for president of the campus organization that you had joined but lost, 2) over lunch one afternoon, you talked to a man or woman you found attractive, but the next afternoon he or she sat at another empty table despite having noticed you, and 3) you visited a professor to discuss a test but he ended the conversation because he was busy. Each video clip lasted for approximately 1–3 min. They were presented on a 1.5 m × 2 m screen placed 3.4 m away from the participant. First, the participants were instructed to watch the video clips passively, imagining that they were experiencing the situations for themselves. Immediately after the offset of each video clip, the participants were asked to rate their current depressive mood on a scale of zero (not at all depressed) to 100 (very depressed) to check if the video clips enhanced their depressive mood. Next, they were asked to generate as many interpretations as possible to reduce the depressive mood. Different interpretations may be generated by the scenarios, and there may be individual differences in which scenarios generate more interpretations in response. Because stress-induced cortisol does not influence recall [20], the same video clips were presented to the participants twice (before and after the stress induction task) in order to control for individual differences.

All responses were coded by two independent coders who specialize in clinical psychology and who were not informed of the study’s hypotheses or the participants’ status. The coding procedure was taken from Wisco and Nolen-Hoeksema [11]. All responses were presented to the coders in random order. The coders rated the negativity of each response using a five-point scale anchored by l = not at all negative and 5 = very negative. Acceptable interrater reliability was obtained from all participants’ interpretations (scenario 1: ICC = .84, scenario 2: ICC = .84, scenario 3: ICC = .94). Three responses were removed as negative interpretations due to their ratings above 3.

### Procedure

**Fig. 1:**
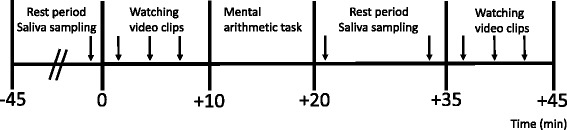
Overview of the experimental procedure

An overview of the experimental procedure is shown in Fig. 1. Experiments began at 1300 h or 1500 h and lasted for approximately 90 min. On arrival, the participants remained seated in a quiet room for 45 min. At the beginning of this rest period, they gave written informed consent, then completed the CES-D. At +15, +30, and +45 min with reference to the onset of the rest period, they completed the PANAS-N and provided a saliva sample. However, we used the cortisol level at +45 min after the onset of the rest period as the baseline cortisol level (T1) because resting for at least 45 min before administering tasks is necessary to control for potential confounders [21]. After the rest period, the participants viewed one practice video clip followed by three measurement video clips, then rated their current depressive mood and generated as many non-negative interpretations as possible. Next, a judge entered the room to administer the mental arithmetic task. After the task, the judge left the room and the participants remained seated for 10 min. At +0 (T2) and +10 min (T3) with reference to the onset of the post-stress rest period, the participants completed the PANAS-N and provided a saliva sample. Because the cortisol level was measured to allocate the participants to either a responder or non-responder group, the timing of post-stress saliva sampling was set to capture a rise in cortisol levels in response to a stressor, based on the report that significant saliva cortisol elevation was observed 1–10 min after a stressor onset, if not peak [22]. After the saliva sampling at T3, the participants again viewed the same video clips that were presented before the stress induction task, followed by rating their current depressive mood and generating as many non-negative interpretations as possible. The post-stress assessment of the generation of non-negative interpretations was conducted from 15 min after the offset of the stress induction task because the relationship between stress-induced cortisol and enhanced emotional processing was found when measured shortly after stress exposure [3]. Finally, the participants were were debriefed about the experiment and paid for their participation.

### Data analyses

**Fig. 2:**
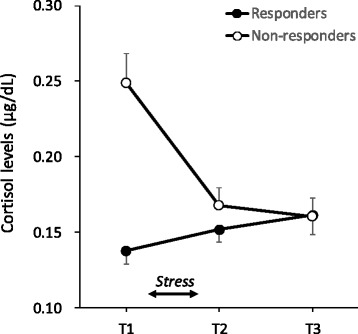
Cortisol levels for the responders and non-responders. Error bars indicate standard error of the mean

The participants were allocated post-hoc to either a responder or non-responder group based on the cortisol response. The participants were allocated to the responder group if their maximum cortisol level during the post-stress period (T2, T3) was higher than the baseline cortisol level (T1). The non-responder group comprised participants who were not included in the responder group. Age, depressive symptoms, and the sex of the responder and non-responder groups were compared by means of the Welch’s unpaired t-tests and Fisher’s exact test, respectively. To assess cortisol and negative affective responses between the responder and non-responder groups, two-way Group (responder, non-responder) × Time (T1, T2, T3) mixed-design analyses of variance (ANOVAs) were used for the cortisol levels and PANAS-N scores. Due to non-normality, log-transformed cortisol values were analyzed. However, raw data are presented in Table 1 and Fig. 2. Furthermore, the percentage increase from the baseline cortisol level (T1) to the maximum values of the cortisol levels after stress (T2, T3) was compared between the groups using a Welch’s unpaired *t*-test. To assess the associations between cortisol response and the generation of non-negative interpretations, a two-way Group (responder, non-responder) × Time (pre-stress, post-stress) mixed-design ANOVA was performed on the number of non-negative interpretations. Because the baseline cortisol level and sex ratio were different between the responder and non-responder groups, a one-way analysis of covariance (ANCOVA) was used, with the number of post-stress non-negative interpretations as a dependent variable, group as an independent variable, and sex, baseline cortisol level, and the number of pre-stress interpretations as covariates. Pearson’s correlation analysis was used to assess the strength of association between change in the cortisol level and change in the number of non-negative interpretations. For all statistical analyses, the significance levels were set to .05 (two-tailed).

**Table 1:** Group characteristics

	Responder (*n* = 19)		Non-responder (*n* = 17)	
	*n*	*%*	*n*	*%*
Sex (Women)	10	53	15	88
	*M*	*SD*	*M*	*SD*
Age	21.3	2.1	21.2	1.4
CES-D	10.37	6.52	12.47	12.01
Baseline cortisol levels (μg/dL)	0.14	0.04	0.25	0.08
Baseline PANAS-N scores	12.63	5.25	10.24	3.47

*Note: CES-D* center for epidemiological studies depression scale

## Results

### Group characteristics

The means and standard deviations (*SD*s) for age, depressive symptoms, baseline cortisol level, and baseline negative affect and sex ratio for the responder and non-responder groups are shown in Table 1. The responder and the non-responder groups were not different in age (*t*(31.7) = 0.05, *n.s.*) or depressive symptoms (*t*(23.6) = 0.63, *n.s.*), but there were more women in the non-responder group (*p* < .05).

### Cortisol response

A two-way Group (responder, non-responder) × Time (T1, T2, T3) mixed-design ANOVA was performed on the log-transformed cortisol level (Fig. 2). The analysis showed that the main effects of group (*F*(1, 34) = 5.18, *p* < .05) and time (*F*(2, 68) = 11.66, *p* < .01) and the interaction (*F*(2, 68) = 43.45, *p* < .01) were significant. A simple effect of time showed that the responders (*F*(2, 68) = 5.29, *p* < .01) and the non-responders (*F*(2, 68) = 49.82, *p* < .01) differentiated among time. For the responders, the cortisol level at T3 was higher than that at T1, and for non-responders, the cortisol levels at T2 and T3 were lower than that at T1 (MSe = 0.004, *p*s < .05). In addition, the baseline cortisol level of the responders was lower than that of the non-responders (*F*(1, 34) = 30.00, *p* < .01). With respect to the percentage increase in cortisol level, the responders had a higher percentage increase in cortisol (*M* = 37.91 %, *SD* = 33.32) than the non-responders (*M* = −21.19 %, *SD* = 12.33) (*t*(23.3) = 7.20, *p* < .01). The results indicate that cortisol elevation was observed only in the responder group.

### Negative affective response

**Fig. 3:**
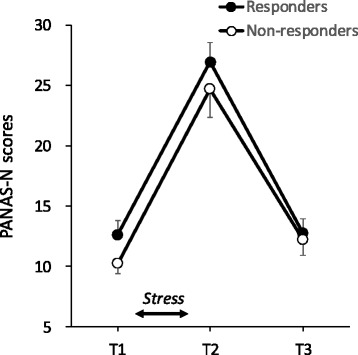
PANAS-N scores for the responders and non-responders. Error bars indicate standard error of the mean

A two-way Group (responder, non-responder) × Time (T1, T2, T3) mixed-design ANOVA was performed on the PANAS-N scores (Fig. 3). The analysis showed that the main effect of time was significant (*F*(2, 68) = 84.14, *p* < .01). the PANAS-N score at T2 was higher than those at T1 and T3 (MSe = 27.46, *p*s < .05). The result indicates that both groups showed increased negative affect in response to the stress induction task.

### Generation of non-negative interpretations

**Fig. 4:**
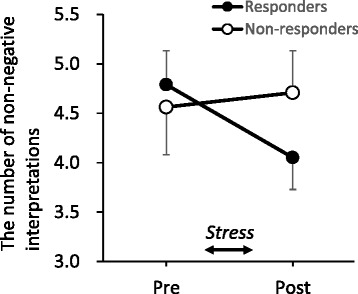
The number of non-negative interpretations for the responders and non-responders. Error bars indicate standard error of the mean

The participants’ mean pre- and post-stress ratings and *SD*s for the mean depressive mood for the three scenarios were 44.75 (*SD* = 17.44) and 44.06 (*SD* = 18.16), respectively. There were no differences in the ratings of mean depressive mood among the three scenarios between the responder and non-responder groups (*t*s = 0.52–1.51, *n.s.*). A two-way Group (responder, non-responder) × Time (pre-stress, post-stress) mixed-design ANOVA was performed on the number of non-negative interpretations generated (Fig. 4). The analysis showed that the interaction was significant (*F*(1, 34) = 7.30, *p* < .05). A simple effect of time showed that only the responders differentiated among time (*F*(1, 18) = 20.10, *p* < .01). For the responders, the number of post-stress non-negative interpretations was lower than the number of pre-stress non-negative interpretations. For the non-responders, the number of non-negative interpretations generated was constant following the stress induction task. A one-way ANCOVA with the number of post-stress interpretations as a dependent variable, group as an independent variable, and sex, baseline cortisol level, and the number of pre-stress interpretations as covariates indicated that the main effects of group were significant (*F*(1, 31) = 4.37, *p* < .05). Correlation analysis revealed no significant correlation between change in the cortisol level and change in the number of non-negative interpretations. These results indicate that the responders showed difficulty in generating non-negative interpretations.

## Discussion

The study tested the hypothesis that stress-induced cortisol would be associated with the generation of fewer non-negative interpretations to depression-related stressful social situations. To test this hypothesis, we compared the change in the number of non-negative interpretations generated following the stress induction task between responders and non-responders. In responders, the number of non-negative interpretations generated decreased after the stress induction task, whereas in non-responders the number of non-negative interpretations generated was constant. Furthermore, the number of post-stress non-negative interpretations was lower for the responders than the non-responders when compared by sex, baseline cortisol level, and the number of pre-stress non-negative interpretations, statistically controlled. These results support the hypotheses. However, inconsistent with the hypothesis, our results found no significant correlation; this was probably partly due to the small sample size. Thus, the results, although inconclusive, suggest the possibility that stress-induced cortisol might be related to decreased generation of non-negative interpretations during cognitive reappraisal of depression-related stressful social situations.

A plausible mediator between increased stress-induced cortisol and a decrease in the number of non-negative interpretations generated following the stress induction task could be enhanced attentional bias toward a neutral stimulus. It has been reported that stress-induced cortisol is related to attentional bias toward negative stimuli [3]. Attentional bias is reported to be associated with cognitive reappraisal. For instance, an eye tracking study reported that, during cognitive reappraisal, attention is more frequently oriented toward non-emotional elements of threatening stimuli, and less frequently toward negative elements [23]. Generating non-negative interpretations requires the scanning not only of negative but also of non-negative stimuli. Due to limited attentional resources, attentional bias toward negative stimuli enhanced by cortisol elevation might hamper the allocation of attentional resources to non-negative stimuli.

Another possible mediator could be working memory impairment caused by stress-induced cortisol. It is reported that stress-induced cortisol secretion is related to working memory impairment [24, 25]. In addition, it has been reported that the efficacy of cognitive reappraisal in the attenuation of negative affective responses is related to individual differences in working memory [9, 26], and it is assumed that working memory would influence the generation of non-negative interpretations during cognitive reappraisal. However, whether or not working memory is related to the generation of non-negative interpretations has not been investigated. Thus, this interpretation awaits further studies.

The effects of cortisol on emotional information processing depend on the timing of its measurement after stress induction [27]. Cortisol enhances excitability and the onset of the stress reaction from shortly after stress exposure (i.e., fast effects). These fast effects are mediated by non-genomic pathways through membrane-located receptors. Next, approximately 15–30 min after receptor activation, cortisol begins to suppress excitability and the stress reaction (i.e., slow effects). These slow effects are mediated by modification of the transcription of target genes through intracellular receptors [28]. In line with the assumption, cortisol is associated with attentional bias toward negative stimuli when measured shortly after a stress-induction task [3], whereas no such results were obtained when measured at longer intervals after a stress-induction task [29]. Because the present study measured the number of non-negative interpretations generated shortly after the stress-induction task, it could capture the fast effects of cortisol to enhance emotional information processing. The generation of fewer non-negative interpretations, when measured shortly after the stress induction task in the present study, is consistent with these previous findings.

Cortisol responders had a lower baseline cortisol level and included more men. These results align with those of previous studies reporting that the basal cortisol level was negatively correlated with cortisol response [30] and that men show higher cortisol response than women [31, 32]. Blunted cortisol response in individuals with a higher baseline level may be due to the baseline level being close to the stress level or to a ceiling effect [30]. Blunted response in women is presumed to be due to actions of gonadal hormones [33]. Because responders had a lower number of post-stress non-negative interpretations with the baseline cortisol level and sex, statistically controlled, cortisol reactivity could lead to difficulty in generating non-negative interpretations. However, the baseline cortisol level and sex could impact the generation of non-negative interpretations as well as cortisol reactivity. Because individuals with a lower basal cortisol level and male sex indicate higher cortisol response, they would be likely to show changes in emotional information processing and difficulty in generating non-negative interpretations.

It is assumed that cortisol could contribute to the development and maintenance of depression [12]. The present study suggests that higher cortisol stress reactivity or sensitivity might hamper the generation of non-negative interpretations during cognitive reappraisal, which might contribute to the maintenance of depressive symptoms.

This study has several limitations. First, the correlation between change in cortisol and change in the number of non-negative interpretations was not significant; this may be partly due to the small sample size and inadequate power to detect correlations. Second, the sex ratio was different between the responder and non-responder groups. This unbalanced sex ratio may confound the relationship between cortisol and the generation of non-negative interpretations. Third, the non-responder group showed a higher baseline cortisol level than the responder group. The higher baseline cortisol level would not be due to anticipatory anxiety because the pre-stress negative effect was not different between the groups. It has been reported that the basal cortisol level is negatively correlated to cortisol response to a stressor [30], thus the participants with a higher basal cortisol level may have shown lower cortisol response to the relatively mild stressor used in the study.

## Conclusions

The study showed that stress-induced cortisol is associated with difficulty in generating non-negative interpretations of depression-related stressful social situations. The responders generated a lower number of post-stress non-negative interpretations when sex, baseline cortisol level, and the number of pre-stress non-negative interpretations were statistically controlled. Although baseline cortisol and sex may impact the generation of non-negative interpretations as well as cortisol reactivity, the results suggest a relation between stress-induced cortisol and cognitive change, which is a later stage of cognitive emotion regulation. Although the cross-sectional study design cannot prove a causal relation, this might be caused by the cortisol acting to facilitate emotional information processing, which hampers the allocation of attentional resources to non-negative stimuli.

## References

[1] Williams JMG, Watts FN, MacLeod C, Mathews A (1997). Cognitive psychology and emotional disorders.

[2] Wieser MJ, Pauli P, Reicherts P, Mu¨hlberger A (2009). Don’t look at me in anger! Enhanced processing of angry faces in anticipation of public speaking. Psychophysiology.

[3] Roelofs K, Bakvis P, Hermans EJ, van Pelt J, van Honk J (2007). The effects of social stress and cortisol responses on the preconscious selective attention to social threat. Biol Psychol..

[4] Lovallo WR (2005). Stress & health.

[5] McEwen BS (2005). Glucocorticoids, depression, and mood disorders: structural remodeling in the brain. Metabolism..

[6] Gross JJ (1998). Emotion regulation: past, present, future. Cogn Emot..

[7] Ochsner KN, Gross JJ (2005). The cognitive control of emotion. Trends Cogn Sci..

[8] Gross JJ (2002). Emotion regulation: affective, cognitive, and social consequence. Psychophysiology..

[9] McRae K, Jacobs SE, Ray RD, John OP, Gross JJ (2012). Individual differences in reappraisal ability: Links to reappraisal frequency, well-being, and cognitive control. J Res Pers..

[10] Joormann J, Gotlib IH (2010). Emotion regulation in depression: relation to cognitive inhibition. Cognition Emotion..

[11] Wisco BE, Nolen-Hoeksema S (2010). Interpretation bias and depressive symptoms: the role of self-relevance. Behav Res Ther..

[12] Herbert J (2013). Cortisol and depression: three questions for psychiatry. Psychol Med..

[13] Shima S, Shikano T, Kitamura T, Asai M (1985). New self-rating scales for depression. Seishin-Igaku..

[14] Radloff LS (1977). The CES-D scale: a self-report depression scale for research in the general population. Appl Psychol Measurement..

[15] Sato A, Yasuda A (2001). Development of the Japanese version of Positive and Negative Affect Schedule (PANAS) scales. Jpn J Pers..

[16] Watson D, Clark LA, Tellegen A (1988). Development and validation of brief measures of positive and negative affect: the PANAS scales. J Pers Soc Psychol..

[17] Al’Absi M, Bongard S, Buchanan T, Pincomb GA, Licinio J, Lovallo WR (1997). Cardiovascular and neuroendocrine adjustment to public speaking and mental arithmetic stressors. Psychophysiology..

[18] van Stegeren AH, Wolf OT, Kindt M (2008). Salivary alpha amylase and cortisol responses to different stress tasks: impact of sex. Int J Psychophysiol..

[19] Krantz S, Hammen C (1979). Assessment of cognitive bias in depression. J Abnorm Psychol..

[20] Elzinga BM, Bakker A, Bremner JD (2005). Stress-induced cortisol elevations are associated with impaired delayed, but not immediate recall. Psychiat Res..

[21] Kudielka BM, Wüest S, Kirschbaum C, Hellhammer DH, Fink G (2010). The Trier Social Stress Test (TSST). Encyclopedia of Stress.

[22] Dickerson SS, Kemeny ME (2004). Acute stressors and cortisol responses: a theoretical integration and synthesis of laboratory research. Psychol Bull..

[23] van Reekum CM, Johnstone T, Urry HL, Thurow ME, Schaefer HS, Alexander AL, Davidson RJ (2007). Gaze fixations predict brain activation during the voluntary regulation of picture-induced negative affect. Neuroimage..

[24] Elzinga BM, Roelofs K (2005). Cortisol-induced impairments of working memory require acute sympathetic activation. Behav Neurosci..

[25] Stauble MR, Thompson LA, Morgan G (2013). Increases in cortisol are positively associated with gains in encoding and maintenance working memory performance in young men. Stress..

[26] Schmeichel BJ, Volokhov RN, Demaree HA (2008). Working memory capacity and the self-regulation of emotional expression and experience. J Pers Soc Psychol..

[27] Oitzl MS, Champagne DL, van der Veen R, de Kloet RE (2010). Brain development under stress: hypotheses of glucocorticoid actions revisited. Neurosci Biobehav Rev..

[28] de Kloet ER, Karst H, Joëls M (2008). Corticosteroid hormones in the central stress response: quick-and-slow. Front Neuroendocrin..

[29] McHugh RK, Behar E, Gutner CA, Geem D, Otto MW (2010). Cortisol, stress, and attentional bias toward threat. Anxiety Stress Coping..

[30] Kudielka BM, Schommer NC, Hellhammer DH, Kirschbaum C (2004). Acute HPA axis responses, heart rate, and mood changes to psychosocial stress (TSST) in humans at different times of day. Psychoneuroendocrinology..

[31] Kirschbaum C, Kudielka BM, Gaab J, Schommer NC, Hellhammer DH (1999). Impact of gender, menstrual cycle phase, and oral contraceptives on the activity of the hypothalamus-pituitary-adrenal axis. Psychosom Med..

[32] Kudielka BM, Kirschbaum C (2005). Sex differences in HPA axis responses to stress: a review. Biol Psychol..

[33] Paris JJ, Franco C, Sodano R, Freidenberg B, Gordis E, Anderson DA, Forsyth JP, Wulfert E, Frye CA (2010). Sex differences in salivary cortisol in response to acute stressors among healthy participants, in recreational or pathological gamblers, and in those with posttraumatic stress disorder. Horm Behav..

